# Effect of Prebiotics-Enhanced Probiotics on the Growth of *Streptococcus mutans*

**DOI:** 10.1155/2019/4623807

**Published:** 2019-08-01

**Authors:** Santichai Nunpan, Chatrudee Suwannachart, Kornchanok Wayakanon

**Affiliations:** ^1^Department of Restorative Dentistry, Naresuan University, Phitsanulok, Thailand; ^2^Biodiversity Research Centre, Thailand Institute of Scientific and Technological Research, Khlong Luang, Pathum Thani, Thailand

## Abstract

*Streptococcus mutans* predominantly creates an acidic environment in an oral cavity. This results in dental demineralization and carious lesions. The probiotics are beneficial microorganisms that modulate the bacterial balance in the digestive system. Prebiotics are defined as nondigestible oligosaccharides that are utilized for the selective stimulation of the beneficial microorganisms. The objective of this study was to evaluate the efficacy of the prebiotics, galactooligosaccharides (GOS) and fructooligosaccharides (FOS), for enhancing the probiotic *Lactobacillus acidophilus* ATCC 4356, for inhibiting *Streptococcus mutans* (A32-2) for the prevention of dental caries. The growth rate of the *S. mutans* significantly decreased when cocultured with *L. acidophilus* in the GOS-supplemented medium at 3%, 4%, and 5%. In the FOS-supplemented medium, the growth rate of *S. mutans* significantly decreased in all concentrations when cocultured with *L. acidophilus*. There was no significant difference in the growth rate of *L. acidophilus* in all concentrations of either GOS or FOS. It can be concluded that the growth rate of *S. mutans* was significantly retarded when cocultured with *L. acidophilus* and the proper concentration of prebiotics. These prebiotics have potential for a clinical application to activate the function of the naturally intraoral *L. acidophilus* to inhibit *S. mutans*.

## 1. Introduction

Humans are host to oral microbiomes in a positive relationship which is a critical determinant for the regulation of oral symbiosis [1]. The alteration of the oral environment, caused by the frequent consumption of carbohydrates, has been beneficial to acidogenic microorganisms with the consequential creation of a low pH environment in the oral cavity. This altered condition enhances the activities and growth of acidogenic and aciduric microbes, predominantly *S. mutans* and *Lactobacillus* spp. In the homeostasis condition, only 2% of *S. mutans* is contained in the dental biofilm. When the balanced condition is transformed to an acidic environment, the percentage of *S. mutans* and *Lactobacillus* spp. substantially increases [2].

Some strains of *Lactobacillus* spp. are forms of probiotics that are beneficial for balancing the microorganism environment in the gastrointestinal tract [3–5]. However, to be beneficial in the oral cavity, probiotics must firstly aggregate and attach to the oral tissue, which then creates a protective barrier to prevent the colonization of the pathogenic microorganism [6].

The growth and activity of probiotics are enhanced by nondigestible oligosaccharides, namely, prebiotics [6, 7] which are unable to be digested by the host, but do enhance the beneficial effects of probiotics by selectively stimulating the growth and activities of the probiotics [8].

The aims of this study are to investigate the effect of probiotics on *S. mutans* after the probiotics received the prebiotics. The prebiotics in this study, galactooligosaccharides (GOS) and fructooligosaccharides (FOS), have been approved by the FDA.

## 2. Materials and Methods

The *S. mutans* A32-2 is a clinical strain isolated from highly active carious patients [9]. It is a gift from Professor Ian Douglas, University of Sheffield. The probiotic was *L. acidophilus* type strain ATCC 4356^T^ (DSMZ 20079^T^).

### 2.1. Determination of the Culturing Medium for Coculturing

The most appropriate culturing medium was first investigated. *S. mutans* and *L. acidophilus* 10^6^ cells were individually cultured in 3 kinds of culturing media: brain heart infusion (BHI; 3.7% (w/v) BHI powder and 10% (w/v) yeast extract) (Difco Laboratories Inc., Detroit, MI, USA), de Man Rogosa and Sharpe (MRS) (Difco Laboratories Inc., Detroit, MI, USA), and Tryptic soy (TSB; 10% (w/v) Tryptic soy broth with tryptone, 5% (w/v) yeast extract, 1.33% (w/v) KH_2_PO_4_, 2.66% (w/v) K_2_HPO_4_, 0.01% (w/v) MgSO_4_.7H_2_O, 0.01% (w/v) FeCl_2_, 0.01% (w/v) MnSO_4_.4H_2_O, 0.01% (w/v) NaCl, and 0.2% glucose) [10]. All experiments were carried out in 5% CO_2_ at 37°C. The growth patterns of the *S. mutans* and *L. acidophilus* in different culturing media were determined by optical density (OD) measurements at 600 nm and the number of colony forming units (CFU) counted.

### 2.2. Effect of the Prebiotics on *S. mutans* and *L. acidophilus*

An equal number of *S. mutans* and *L. acidophilus* (10^6^ cells) were cocultured in the MRS medium supplemented with 1%, 2%, 3%, 4%, and 5% of GOS (v/v) and FOS (w/v). A control group was formed with the culturing media not supplemented by prebiotics. The effect of the prebiotics on the *S. mutans* and *L. acidophilus* both separately, and as a coculture, was determined by measuring the growth rate at 3, 6, and 12 h. The growth rate was calculated from the formula:

(1) $$\begin{array}{c} \text{growth rate }\left(h^{-1}\right)=\frac{\left[\left(\log_{10}N_{t}-\log_{10}N_{0}\right)\times 2.303\right]}{\left(t-t_{0}\right)}, \end{array}$$

where *N*_*t*_: number of bacteria at time point “*t*,” *N*_0_: number of bacteria at the initial time point (time point “0”), *t*: duration of observing time, and *t*_0_: initial time point (time point “0”).

### 2.3. Effect of Prebiotics on the Cellular Fatty Acids

After coculturing between *S. mutans* and *L. acidophilus* 10^6^ cells each for 6 h, the cellular fatty acid of the *S. mutans* and *L. acidophilus*, separately and cocultured, was analyzed by gas chromatography using the MIDI Sherlock™ microbial identification system. Bacterial pallets 0.06 g or supernatant 0.5 ml from the samples were independently vigorously mixed with 1 ml of Reagent 1 (3.75 N NaOH in deionized water and methanol) for 10 s, and the solutions were then heated in 100°C water for 25 min and then plunged into an ice bath for chilling. For the methylation, 2 ml of Reagent 2 (6.0 N HCl in methanol) was vigorously mixed with the cold sample for 10 s. The samples were then incubated in 80°C water for 10 min and cooled in ice.

For the fatty acid extraction, 1.25 ml of Reagent 3 (hexane: methyl tert-butyl ether (1 : 1 v/v)) was added and consecutively mixed for 10 min. The upper part of the solution was collected and mixed with Reagent 4 (0.3 N NaOH) for 5 min, and the supernatant collected for the fatty acid analysis.

## 3. Results

### 3.1. Suitable Culturing Medium for the Coculture of *S. mutans* and *L. acidophilus*

**Figure 1:**
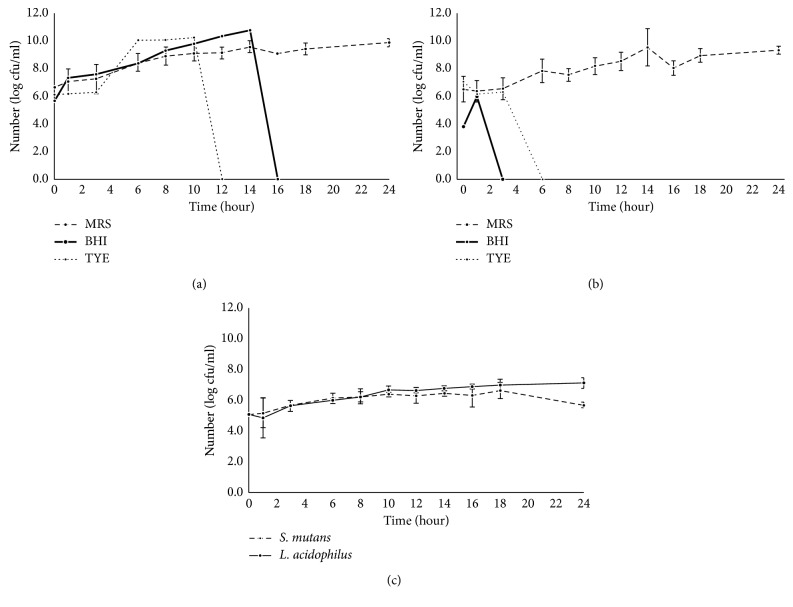
Growth patterns of *S. mutans* (a) and *L. acidophilus* (b) in MRS, BHI, and TYE media. The growth pattern of the *S. mutans* and *L. acidophilus* coculture in the MRS medium (c).

*S. mutans* could survive for 14 h in the BHI and 10 h in the TYE media, and the *S. mutans* grew in the MRS culturing medium throughout the 24-hour growth phase. *L. acidophilus* grew in the TYE for only three hours and only one hour in the BHI medium but grew in the MRS medium with similar patterns as *S. mutans* (see Figures 1(a) and 1(b)). Both *S. mutans* and *L. acidophilus* grew well in the MRS medium. Therefore, the MRS medium was selected for the coculture between *S. mutans* and *L. acidophilus*.

During the first 8-hour period in the MRS medium, the number of viable bacteria in each was similar, but for the subsequent 16 h, the number of *L. acidophilus* consistently exceeded that of *S. mutans* (see Figure 1(c)).

### 3.2. Determination of the Influence of Prebiotics Supplementation on pH

Organic acids are one of the common metabolic products from both *S. mutans* and *L. acidophilus.* In the individual culture, the pH of the FOS-supplemented medium was insignificantly higher than that of GOS-supplemented medium for both *S. mutans* and *L. acidophilus* (*P* > 0.05). In the coculture, there was also no statistical difference in the pH between GOS- and FOS-supplemented media (*P* > 0.05). The pH of the individual *S. mutans* culture (pH = 5.9–6.2) was higher than that of either the individual *L. acidophilus* (pH = 5–5.4) culture or the coculture (pH = 5.1–5.4) (see Table 1).

### 3.3. Effect of the Prebiotics on the Growth of *S. mutans* and *L. acidophilus*

The individual cultures of *S. mutans*, grown in the prebiotics-free medium, showed a growth rate of 0.9921 ± 0.14 h^−1^. When supplemented with the different concentrations of GOS, the growth rates of the individual cultures of *S. mutans* were similar to those of the control group (*P* > 0.05). The most significant decrease in the growth rate of *S. mutans* was observed in 3% FOS as compared to the control (*P*=0.032) (see Table 2).

For the cocultivation in the GOS-supplemented medium, the growth rate of *S. mutans* in the 3% GOS (0.3775 ± 0.06 h^−1^), 4% GOS (0.4672 ± 0.12 h^−1^), and 5% GOS (0.5491 ± 0.09 h^−1^) was significantly decreased compared to the control (0.9623 ± 0.17 h^−1^) (*P* < 0.05) (see Table 2).

When the FOS was added into the coculture, the growth rate of *S. mutans* significantly decreased in all concentrations (*P*=0.034) as compared to the control. The minimum growth rate was found in the 3% FOS (0.2281 ± 0.12 h^−1^) (see Table 2).

For *L*. *acidophilus*, the growth rate of both the individual and cocultures in all concentrations of the GOS- and FOS-supplemented media was insignificantly different from the control (*P* > 0.05) and among the concentrations (see Table 3).

### 3.4. Effect of Prebiotics on the Cellular Fatty Acids

In this study, the cellular fatty acids and the secreted fatty acids were observed in the coculture after being cultured for 6 h.

When *S. mutans* and *L. acidophilus* were grown in the prebiotics-supplemented culturing medium, the percentage of cellular fatty acid was similar to that of the control group. For the cocultures, the percentages of cellular saturated fatty acids and unsaturated fatty acid from the mixed pellets were also not different from those of the control group (see Table 4).

When *S. mutans* and *L. acidophilus* were individually grown or cocultured in the prebiotics-supplemented culturing media, the percentage of secreted fatty acid was similar to the control group (see Table 5).

## 4. Discussion

Dental caries is a major oral problem in all ages. *S. mutans* are the vital cariogenic bacteria, metabolizing sugar with their acidogenic and aciduric properties. Several studies have been reported that attempted to modify the *S. mutans* pathogenicity by adding probiotics such as *L. acidophilus*, suggesting that probiotics could generate a protective barrier, inhibit pathogenic microorganisms, and stimulate the host defensive mechanisms [11]. *L. acidophilus* is also naturally found in small amounts in the oral cavity [12]. The oral probiotics must be able to adhere and to colonize the oral tissue and to compete with pathogenic microorganisms at the binding site [13].

*Lactobacillus* spp. are well-known probiotics which have the ability to adhere to epithelial cells [14] and produce several bacteriocin effects on other microorganisms [15]. *L. acidophilus* (ATCC4356^T^ = DSMZ 20079^T^) was the probiotic applied in this study, as it is the type strain most found in the human body and is normally about 3%–9% of the bacteria found in oral cavities. It has also been previously reported as the preferred probiotics in many studies [16].

For this experiment, the two distinct strains *S. mutans* and *L. acidophilus* were used and cultured in the same media. First, however, it was important that the appropriate culturing conditions for both strains be determined. Based on previous studies, the appropriate culture media for *L. acidophilus* are MRS media and BHI [17, 18] and tryptic soy are suitable for *S. mutans* cultivation [19]. *L. acidophilus* are anaerobic bacteria and require an oxygen-free condition for propagation. However, *L. acidophilus* can be cultured in 5% CO_2_ at 37°C [20]. According to many studies, the interaction between *S. mutans* and *L. acidophilus* occurs in the agar diffusion process under anaerobic conditions [18, 21]. For this study, *S. mutans* and *L. acidophilus* were cocultured in culturing broth under conditions of 5% CO_2_. The results showed that *S. mutans* seemed to be able to grow in almost all types of culturing media. However, the *S. mutans* receded from the TYE or BHI cultures. *L. acidophilus*, which are fastidious microorganisms, need more complex nutrients such as amino acids and peptides [22, 23], trace elements [24], vitamins, oleic acids [25], buffering agents, and polysorbates, for growing and cell activity. All these substances are in the MRS, but not in the BHI and TYE.

According to Singh et al., when the subjects consumed probiotic ice cream containing freeze-dried *L. acidophilus* La5, the number of salivary mutans streptococci significantly decreased [26]. Moreover, *L. acidophilus* DSM 20079^T^ suppressed the ability of *S. mutans* ATCC 35668 to adhere to the microtiter plates, indicating that when *L. acidophilus* were introduced into the system before the *S. mutans* were added, the suppression of salivary mutans streptococci was more efficient [27]. The *L. acidophilus* DSM 20079^T^ also has the ability to decrease the viable count of *S. mutans* [28, 29].

This is the first study to determine the growth rate of *S. mutans* when cocultured with *L. acidophilus* in the prebiotics-supplemented media. Other previous studies only determined the viable count of *S. mutans*, not the growth rate [28, 29]. The bacterial growth rate reflects the ability of bacteria to multiply. In order to simulate the oral situation by applying prebiotics before bed, a six-hour incubation period was selected for determining the growth rates.

After GOS was added into the individual culture, the growth rate of *S. mutans* was not statistically different from the control. However, when the *S. mutans* was cocultured with *L. acidophilus*, the growth rate of the *S. mutans* significantly decreased in the 3%, 4%, and 5% concentrations of GOS as compared to the control group (no prebiotics). It is indicating that the 3% GOS was the optimum concentration that could decrease the growth rate of *S. mutans* when cocultured with *L. acidophilus*.

The growth of *S. mutans* decreased after receiving the FOS, in both the individual culture and the coculture. The minimum growth rate was seen in the 3% FOS in both the individual culture and the coculture. In the coculture, the growth rate in all concentrations decreased, while in the single culture, only the 3% FOS resulted in a decrease. These results indicate that the efficacy of FOS to decrease the growth rate of *S. mutans* was potentiated when *L. acidophilus* was present in the culture.

Even though *L. acidophilus* can utilize both GOS [30] and FOS [31], there is the limitation in GOS digestion, resulting in the metabolized and acidic products being limited [30]. According to this study, the growth rates of *L. acidophilus* were not affected by GOS and FOS in both the individual culture and the coculture. When *L. acidophilus* is cocultured with *S. mutans* in the prebiotic-supplemented media, the growth rates of *S. mutans* decreased. This finding indicates that GOS and FOS might enhance the cell activity of *L. acidophilus* to reduce the growth of *S. mutans*. However, the mechanism of GOS and FOS dominated by *L. acidophilus* has not been identified and needs further studies.

GOS and FOS are resistant to gastric digestion in humans. The energy from GOS is 1.7 kcal/g and from FOS is 1.5 kcal/g, indicating that they can be used as sweeteners in food and beverages. However, excessive intake probably results in abdominal discomfort, flatulence, and diarrhea [32]. The recommended dose ranges between 8 and 20 g/d.

A lipid bilayer is the main composition of the cell membrane. Its components are affected by the composition of the fatty acids. The saturated fatty acid chains, which are less than 12 carbon atoms, have been detected in small amounts in bacteria. The bigger saturated fatty acids, more than 16 carbon atoms, have been found in larger proportions than the small fatty acids, and so the unsaturated fatty acids are a major portion of all the fatty acids of the bacteria. The culturing conditions have an influence on the rearrangement of the membrane fatty acids [33].

*S. mutans* altered their membrane fatty acids during growth in low pH conditions. At pH 6.5, the cellular fatty acids of *S. mutans* UA159 were composed of 65.4% saturated fatty acids and 34.6% unsaturated fatty acids. When the pH drops to 5.5, the saturated fatty acids reduced to 53.3% and the unsaturated fatty acids increased to 46.7% [34]. Bender et al. mentioned that the unsaturated fatty acids of *S. mutans* increased in acidic conditions especially for C18 : 1 and C20 : 1 [35].

In 1991, Sato et al. found that the lipids composition of the cell membrane of *S. mutans* markedly changed when fatty acids were added into the growth medium [36]. Even though the fatty acids are the cell composition, they have an antimetabolite effect on several microorganisms [37]. The half-time for pH equilibration of *S. mutans* significantly increased when lauric acid and palmitic acid were added, but it slightly increased when arachidic acid was added [38]. Carson and Daneo-Moore found that the oleic acid was able to induce the lysis of bacterial membrane due to the susceptibility of the double bond of unsaturated fatty acids to the oxidizing agent [39]. In this study, the growth rate of *S. mutans* in 3% FOS significantly decreased compared with the other groups, but there was no significant difference on each kind of fatty acids in that group. The prebiotics had no effect on the fatty acid composition of the cell membrane.

The results from this study showed that GOS and FOS, which are polysaccharide prebiotics, do not support the growth rate of *S. mutans* and *L. acidophilus*. On the contrary, the 3% FOS decreased the growth rate of *S. mutans*. When considering the clinical applications, the 3% FOS might be useful as a treatment for high caries-risk patients to reduce the growth rate of *S. mutans* in the oral cavity. In addition, the 3%, 4%, and 5% of GOS and 1%, 2%, 3%, 4%, and 5% of FOS have potential as a clinical application to activate the function of the intraoral *L. acidophilus*, which is the naturally occurring probiotic, resulting in a decrease in the growth rate of *S. mutans*, but with no increase in the number of cariogenic bacteria. However, the mechanism of action is obscure. To identify the specific mechanism, further studies are required.

## 5. Conclusions

The prebiotics, GOS and FOS, had no effect on the growth rates of *L. acidophilus* ATCC 4356, and the 3% FOS significantly decreased the growth rate of *S. mutans* A32-2. The 3% concentration of GOS and the 1% of FOS seem to have the potential to decrease the growth rate of *S. mutans* when applied together with the probiotics *L. acidophilus*.

## Figures and Tables

**Table 1:** pH of the culturing medium when cultured for 6 h.

Prebiotic concentrations (%)	Individual culture				Coculture	
	*S. mutans*		*L. acidophilus*		*S. mutans* and *L. acidophilus*	
	GOS	FOS	GOS	FOS	GOS	FOS
0	5.98 ± 0.12^A^		5.19 ± 0.31^A^		5.34 ± 0.01^A^	
1	5.95 ± 0.11^A,a^	6.16 ± 0.02^A,a^	5.04 ± 0.22^A,a^	5.32 ± 0.33^A,a^	5.38 ± 0.39^A,a^	5.34 ± 0.31^A,a^
2	6.01 ± 0.05^A,a^	6.21 ± 0.02^A,a^	5.11 ± 0.17^A,a^	5.39 ± 0.35^A,a^	5.32 ± 0.57^A,a^	5.32 ± 0.30^A,a^
3	5.96 ± 0.09^A,a^	6.21 ± 0.10^A,a^	5.07 ± 0.23^A,a^	5.38 ± 0.29^A,a^	5.31 ± 0.24^A,a^	5.33 ± 0.51^A,a^
4	5.94 ± 0.05^A,a^	6.16 ± 0.10^A,a^	5.00 ± 0.20^A,a^	5.34 ± 0.36^A,a^	5.09 ± 0.22^A,a^	5.31 ± 0.33^A,a^
5	5.90 ± 0.19^A,a^	6.16 ± 0.11^A,a^	5.11 ± 0.28^A,a^	5.38 ± 0.42^A,a^	5.10 ± 0.48^A,a^	5.28 ± 0.35^A,a^

*Note.* The statistics compared within each type of prebiotics. The uppercase letters indicate the significant difference within each column. The lowercase letters indicate the significant difference within each row.

**Table 2:** Growth rate of *S. mutans* grown individually and cocultured in different concentrations of prebiotics in the MRS medium.

Prebiotic concentration (%)	GOS (%v/v)		FOS (%w/v)	
	Individual culture	Coculture	Individual culture	Coculture
0 (control)	0.9921 ± 0.14^A,a^	0.9623 ± 0.17^A,a^	0.9921 ± 0.14^X,x^	0.9623 ± 0.17^X,x^
1	1.0058 ± 0.13^A,a^	0.8391 ± 0.19^A,a^	0.5419 ± 0.01^X,x^	0.5209 ± 0.09^Y,x^
2	0.9099 ± 0.29^A,a^	1.0272 ± 0.01^A,C,a^	0.6467 ± 0.07^X,x^	0.5683 ± 0.10^Y,x^
3	0.7104 ± 0.02^A,a^	0.3775 ± 0.06^B,a^	0.2998 ± 0.11^Y,x^	0.2281 ± 0.12^Z,x^
4	1.1588 ± 0.37^A,a^	0.4672 ± 0.12^B,a^	0.5604 ± 0.07 ^X, x^	0.5724 ± 0.06^Y,x^
5	0.9070 ± 017^A,a^	0.5491 ± 0.09^B,a^	0.7123 ± 0.06^X,x^	0.3429 ± 0.07^Z,x^

*Note.* The statistics compared within each type of prebiotics. The uppercase letters indicate the significant difference within each column. The lowercase letters indicate the significant difference within each row.

**Table 3:** Growth rate of *L. acidophilus* grown individually and cocultured in different concentrations of prebiotics in the MRS medium.

Prebiotic concentration (%)	GOS (%v/v)		FOS (%w/v)	
	Individual cultured	Cocultured	Individual cultured	Cocultured
0 (control)	0.6446 ± 0.06^A,a^	0.5743 ± 0.30^A,a^	0.6446 ± 0.06^X,x^	0.5743 ± 0.30^X,x^
1	0.3379 ± 0.01^A,a^	0.7895 ± 0.26^A,a^	0.4703 ± 0.29^X,x^	0.4322 ± 0.20^X,x^
2	0.4301 ± 0.19^A,a^	0.7691 ± 0.39^A,a^	0.5287 ± 0.23^X,x^	0.6357 ± 0.08^X,x^
3	0.4065 ± 0.04^A,a^	0.6268 ± 0.18^A,a^	0.4802 ± 0.07^X,x^	0.6580 ± 0.13^X,x^
4	0.5049 ± 0.27^A,a^	0.6583 ± 0.25^A,a^	0.6848 ± 0.12^X,x^	0.4625 ± 0.13^X,x^
5	0.6796 ± 0.08^A,a^	0.5806 ± 0.07^A,a^	0.3546 ± 0.01^X,x^	0.3971 ± 0.09^X,x^

*Note.* The statistics compared within each type of prebiotics. The uppercase letters indicate the significant difference within the columns. The lowercase letters indicate the significant difference within the rows.

**Table 4:** Percentages of cellular fatty acids of individual *S. mutans* and *L. acidophilus* and the cocultured in prebiotics-supplemented media.

Percentage of prebiotics		Single culture				Coculture	
		*S. mutans*		*L. acidophilus*		*S. mutans/L. acidophilus*	
		Unsaturated	Saturated	Unsaturated	Saturated	Unsaturated	Saturated
Control (MRS)		55.81	44.19	72.45	27.55	80.05	19.95

GOS	1(%)	57.43	42.57	78.36	21.64	81.59	18.41
	2(%)	52.47	47.53	77.50	22.50	82.36	17.64
	3(%)	52.38	47.62	77.45	22.55	82.67	17.33
	4(%)	51.15	48.85	76.61	23.39	84.61	15.39
	5(%)	43.55	56.45	79.34	20.67	84.99	15.01

FOS	1(%)	57.55	42.45	76.04	23.96	80.80	19.20
	2(%)	58.41	41.59	75.91	24.09	80.96	19.04
	3(%)	59.31	40.69	76.80	23.20	82.62	17.38
	4(%)	57.61	42.39	78.93	21.07	79.37	20.63
	5(%)	54.74	45.26	78.53	21.47	85.25	14.75

**Table 5:** Percentages of secreted fatty acids of individual *S. mutans* and *L. acidophilus* and the cocultured in prebiotics-supplemented media.

Percentage of prebiotics		Single culture				Coculture	
		*S. mutans*		*L. acidophilus*		*S. mutans/L. acidophilus*	
		Unsaturated	Saturated	Unsaturated	Saturated	Unsaturated	Saturated
Control (MRS)		75.52	24.48	75.24	24.76	75.05	24.95

GOS	1(%)	75.25	24.75	74.01	25.99	75.04	24.96
	2(%)	52.48	47.52	73.56	26.40	73.94	26.06
	3(%)	73.43	26.57	73.71	26.29	72.79	27.21
	4(%)	69.02	30.98	72.95	27.05	73.25	26.75
	5(%)	75.38	24.62	72.78	27.22	74.70	25.30

FOS	1(%)	75.58	24.42	74.41	25.59	72.15	27.85
	2(%)	77.74	22.26	75.37	24.63	72.94	27.06
	3(%)	76.19	23.81	75.39	24.61	74.31	25.69
	4(%)	76.29	23.71	74.91	25.09	73.27	25.23
	5(%)	78.85	24.15	75.27	24.73	73.87	26.13

## Data Availability

The data used to support the findings of this study are included within the article.
